# Children’s color-based perception of warning intensity in traffic safety signs

**DOI:** 10.3389/fpsyg.2026.1866078

**Published:** 2026-10-08

**Authors:** Jiaqi Pang, Chenhao Pang, Luwei Chen

**Affiliations:** 1 School of Advanced Manufacturing, China Jiliang University College of Modern Science and Technology, Jinhua, China; 2 School of Design, The Hong Kong Polytechnic University, Hong Kong, Hong Kong SAR, China

**Keywords:** children, children’s color perception, icon design, traffic safety signs, warning color

## Abstract

Children’s perceptions of warning intensity in traffic safety signs have received limited empirical attention, despite their importance for child-oriented safety design. Combining structured color-selection tasks with qualitative interviews, this study examines how children interpret warning intensity conveyed by color and whether these interpretations vary with sign shape or situational context. Thirty children aged 11–12 participated in a gamified task in which they assigned colors to five warning levels across multiple frame-icon combinations. Their color choices were analyzed in the CIELCH color space and interpreted together with interview responses. Differences in L* and C* across sign shapes and traffic scenarios were examined using Friedman tests, followed by paired Wilcoxon signed-rank tests. Differences in *hab* were examined using repeated-measures circular MANOVA, followed by paired two-condition circular MANOVA tests. The quantitative results were interpreted together with the findings from the qualitative interviews. The results show that children consistently associated red with the strongest warnings and yellow with strong warnings, largely regardless of sign shape or traffic context. Compared with adult-based standards, children selected colors with greater lightness at the highest warning level, while their lightness and chroma distributions at the two highest warning levels showed deviations and heterogeneity. The interviews further suggest that judgments of warning intensity are driven primarily by color salience, whereas shape and imagery play more limited roles. These findings highlight the importance of incorporating children’s perceptual characteristics into the design of traffic-safety signs and warning displays, which have traditionally been based on adult perceptions.

## 1 Introduction

Traffic signs play a critical role in ensuring the safety and efficiency of modern transportation systems. Among them, warning signs are specifically designed to capture attention and convey potential hazards, thereby enabling road users to take timely and appropriate action (Wogalter et al., 1999; Shinar, 2017). The effectiveness of traffic warning signs relies heavily on their ability to deliver clear, rapid, and universally understood messages to all road users, particularly pedestrians, who are more directly exposed to hazards in the traffic environment (Yao et al., 2019). Consequently, the design of such signs has become a crucial aspect of transportation safety engineering and visual communication design (Dewar and Olson, 2007; Ng and Chan, 2007).

A typical traffic warning sign combines three essential visual features, including color, pictorial iconography, and geometric shape, to ensure rapid and accurate recognition (International Organization for Standardization, 2011; Federal Highway Administration, 2009). Color is perhaps the most immediate perceptual cue, with specific hues (e.g., red and yellow) conventionally associated with danger, caution, or prohibition (Wogalter et al., 2002; Braun and Silver, 1995). The iconography of a sign varies across contexts, for example by depicting vehicles, bicycles, or pedestrians, and is tailored to signal the relevant hazard type (Ng and Chan, 2007). Finally, the geometric shape of a sign’s frame (such as triangles for warnings, circles for regulations, or diamonds for general caution) provides an additional coding layer that enhances rapid recognition and compliance (Shinar, 2017).

However, the design of traffic warning signs has historically focused on adult road users. International standards such as ISO 3864-1 (2011) are grounded in adult cognitive processing and risk perception, assuming adults can swiftly interpret warning messages (International Organization for Standardization, 2011). This design paradigm, while effective for the general population, may not adequately consider children’s developmental and perceptual limitations. Children commonly act as independent road users, including pedestrians and cyclists, yet their traffic cognition and perceptual maturity differ significantly from adults (Rothengatter, 1984). Empirical studies have documented that children’s comprehension of road signs varies with age and that they often misinterpret signs designed for adults, as shown in drawing-based evaluations (Siu et al., 2015). A pilot study of 7–11-year-olds in China similarly found generally low levels of traffic sign comprehension (Han et al., 2024), highlighting a notable gap in research on the effectiveness of warning signs for children.

Given that existing standards have largely been developed with adult road users in mind (International Organization for Standardization, 2011), a critical question arises regarding their suitability for children, who process visual warnings differently because of developmental factors (Biassoni et al., 2018; Trifunović et al., 2017; Oxley et al., 2007). Within this context, color deserves particular attention. Empirical evidence shows that color is a highly salient and effective element in safety communication, enhancing attention capture and hazard recognition (Wogalter, 2018). Developmental studies further indicate that children and adults differ significantly in how they perceive, interpret, and emotionally react to colors (Pitchford and Mullen, 2005; Burkitt et al., 2003). For example, while red may typically signal danger to adults, children’s interpretations may vary based on their prior experience and cognitive development.

Moreover, children’s perception of color in traffic signs is closely intertwined with other visual elements, such as shape and pictorial context. Developmental studies suggest that younger children often rely more heavily on concrete pictorial content when interpreting visual symbols, whereas abstract features, such as geometric outlines, may be overlooked or misinterpreted (Uttal and Yuan, 2014). For example, triangular shapes, which are widely recognized by adults as indicators of warning, may not automatically convey the same associations to younger children because of their still-developing symbolic understanding (Siu et al., 2015). Further research has shown that children’s ability to recognize geometric shapes varies across different geometric forms and could also be influenced by developmental and contextual factors (Trifunović et al., 2024). Moreover, a recent study focused on children’s visual-art appreciation found that their observational and interpretive performance varied according to the educational materials and visual contexts presented (Gumulya and Gultom, 2024). Together, these findings raise a question regarding the extent to which children’s perception of the warning function of color may depend on the accompanying pictorial scene and geometric shape of a traffic sign.

Addressing this question provides both theoretical and practical contributions. Theoretically, it extends research on child cognition and visual communication by integrating insights from developmental psychology, human factors, and safety design (Schwebel and Gaines, 2007; Barton and Morrongiello, 2011). Practically, it informs the design of traffic warning signs that account for children’s perceptual and cognitive needs, thereby enhancing road safety for young pedestrians and cyclists. Against this backdrop, the present study aims to investigate the following research questions:

- Shape-related influence: How does the shape of a traffic warning sign interact with color in shaping children’s warning perception?
- Scene-related influence: To what extent does the pictorial content of a traffic warning sign (e.g., car, bicycle, pedestrian) affect children’s perception of the warning effectiveness of color?
- Comparison with standardized colors: How do children’s color-based perceptions of warning intensity compare with internationally standardized warning-color reference values developed primarily for adult-oriented safety contexts?

Based on the preceding literature and research questions, this study proposes the following hypotheses concerning the effects of sign shape and traffic scenario on children’s warning-color selections, as well as their differences from adult-oriented standards:

- *H1*: At the same warning-intensity level, children’s color selections differ across traffic sign shape conditions.
- *H2*: At the same warning-intensity level, children’s color selections differ across traffic scenario conditions.
- *H3*: Children’s warning-color selections will show deviations from adult-oriented internationally standardized warning-color reference values.

These hypotheses provide the basis for the experimental design and data analyses presented in the following sections. Thus, by systematically investigating the research questions and testing the corresponding hypotheses, this study makes three contributions. First, it identifies cognitive and perceptual differences between children and adults in processing color within traffic warning signs. Second, it extends the literature on multimodal sign design by clarifying the interplay between color, scene, and shape in children’s warning perception. Third, it provides evidence-based insights for traffic safety policy and practice, advocating for child-centered approaches in the standardization of traffic sign systems. Ultimately, while international standards serve adult populations effectively, they may fall short for younger users, highlighting the need for design strategies that explicitly incorporate developmental perspectives.

## 2 Materials and methods

This study employed a gamified procedure to help children understand the tasks easily and engage with the traffic-warning perception and color-selection process. The study mainly employed a within-subjects experimental design. We collected both quantitative color-selection data and qualitative semi-structured interview data from the participants. Overall, the study adopted a mixed-methods approach that integrated quantitative and qualitative methods.

### 2.1 Participants recruitment

Primary school children are required to have received education on traffic signs in Grade 3 of primary school (State Council of China, 2007). Thus, the initial inclusion criteria cover both boys and girls aged 9–12 years, generally corresponding to Grades 3–5 of primary school. Further inclusion criteria required participants to have normal or corrected-to-normal vision, including children with mild myopia or astigmatism. Children with color vision deficiencies, severe visual impairments, or other ocular diseases were excluded to ensure accurate perception of the experimental stimuli. To minimize variation in educational background and local living context, all children were recruited from the same primary school. Moreover, an Ishihara’s Test with 24 plates (Royer et al., 2018) was conducted for each participant to ensure the color discrimination ability. Written informed consent was obtained from parents or legal guardians, and verbal or written assent was obtained from all child participants prior to the experiment.

### 2.2 Stimuli and apparatus

Stimuli. The stimuli consisted of digital traffic signs presented through a gamified iPad interface. The study used nine sign-frame combinations, including three sign types (traffic warning scenarios): “Caution: Non-Motor Vehicles,” “Caution: Pedestrians,” and “Caution: Motor Vehicles.” Each sign type was paired with three frame shapes: square, circular, and triangular, as shown in Figure 1.

**FIGURE 1:**
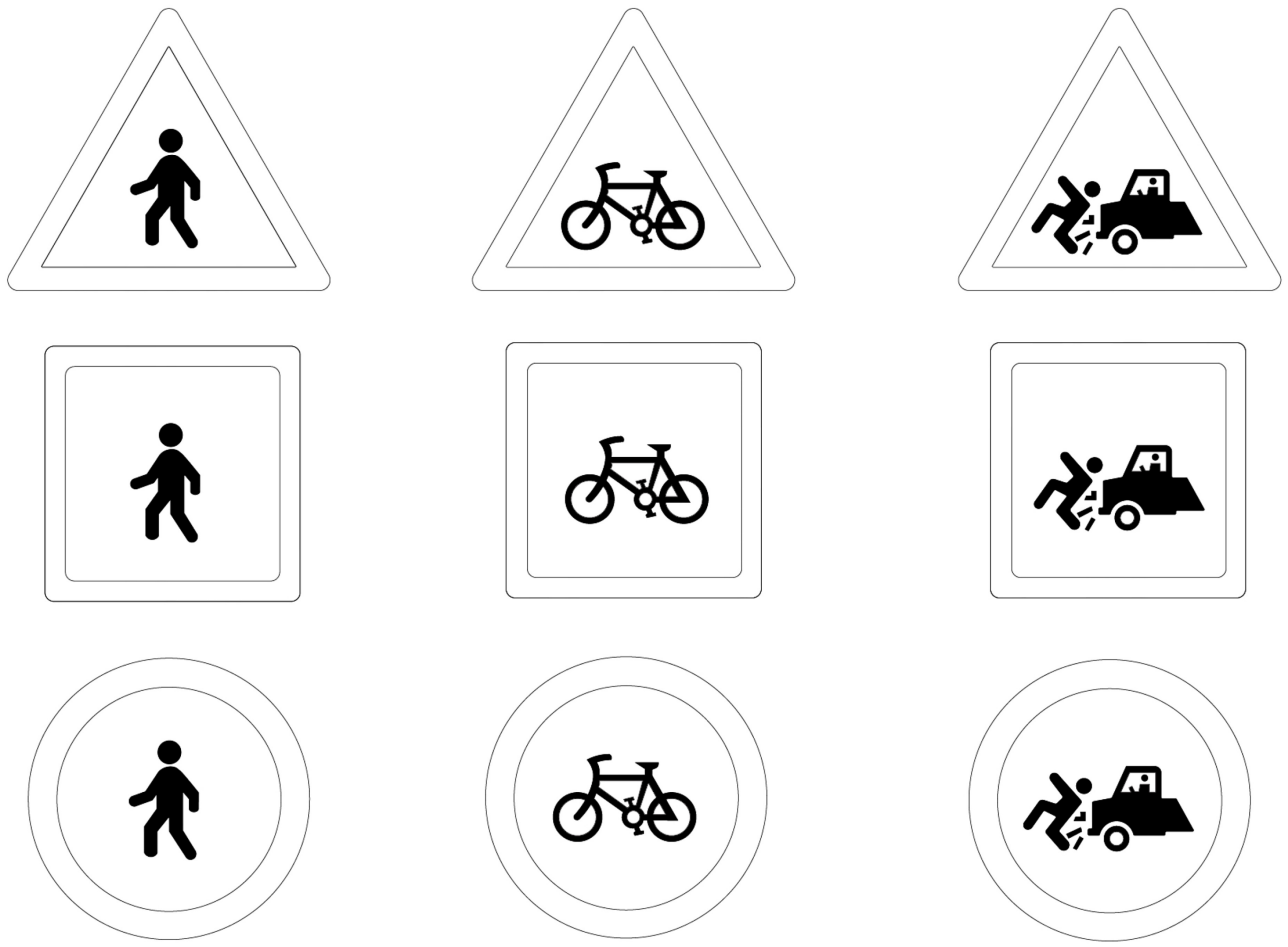
Nine traffic sign-frame combinations.

Each traffic sign was presented with five warning levels, including *No warning, Weak warning, Moderate warning, Strong warning*, and *Very strong warning*, arranged from left to right. This results in a total of 45 coloring tasks per participant. Participants were provided with a color selection interface that included:

- Recommended color palette: A circular color ring with pre-selected colors associated with standard warning levels.
- Free color picker: A digital color palette allowing participants to select any color outside the recommended palette.

Each icon was designed to be visually distinct and recognizable for children, with high-contrast colors, clear borders, and simplified pictograms suitable for the target age group (see Figure 1). To enhance engagement and sustain attention, the iPad-based experiment incorporated gamified elements throughout the task. These included a neutral guide sprite that offered gentle, non-directive instructions; a role-playing component in which participants acted as “junior traffic sign designers”; and reward mechanisms such as digital badges granted upon task completion. The overall game-based stimulus design is shown in Figure 2.

**FIGURE 2:**
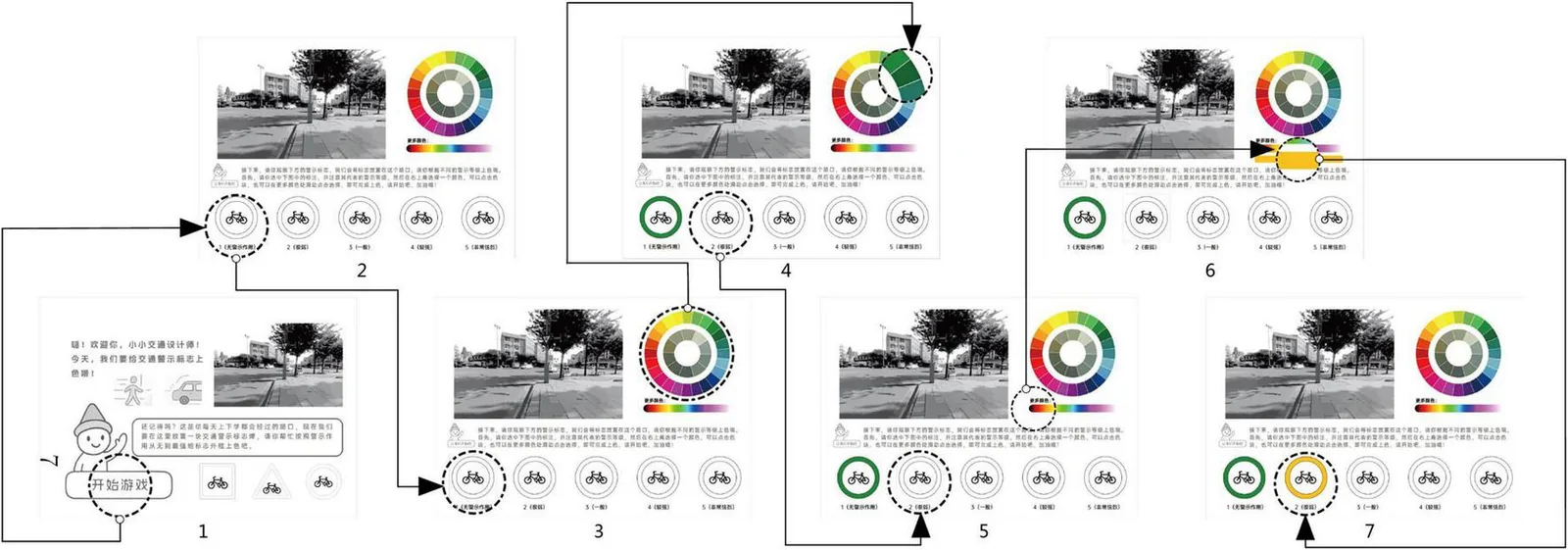
Overview of the stimulus game design: (1) click “Start Game”; (2) select the sign to be colored; (3) confirm that the sign is highlighted; (4) choose a color to fill the sign; (5) select another sign to activate it; (6) use the “More Colors” bar to access the full color range; and (7) apply the selected color to the sign.

Apparatus. All tasks were presented on iPad tablets (Apple iPad Pro 11 with iPadOS 16). The game interface was custom-developed for this study, supporting touchscreen color selection, gamification elements, and data logging of participant interactions, including time spent per task, color changes, and interaction patterns.

### 2.3 Procedures

The experiment was conducted in five phases, as outlined below.

*Step 1: introduction and ethical briefing:* Participants were introduced to the “Little Traffic Designer” role and the accompanying guide sprite. Researchers explained the task objectives, the concept of warning levels (using numerical or emoji representations), and the consent procedures. Children were reminded that the game focused on completing the tasks rather than selecting “correct” colors.

*Step 2: practice session:* The operator instructed the children participant to select colors based on their subjective impressions rather than careful deliberation. Then, participant completed several practice coloring tasks to familiarize themselves with the iPad interface, including the recommended color ring and free color picker. Furthermore, the experimenter confirmed that all child participants correctly understood the meanings of the traffic signs shown in Figure 1. The *guide sprite* demonstrated neutral operational prompts, such as pointing to the color palette and indicating how to switch colors, without suggesting any specific choice.

*Step 3: main gaming task:* Participants completed 45 experimental tasks organized into three blocks of 15 tasks, with each block corresponding to one of the three scenarios described in section 2.2. Participants took a 2-min break between blocks.

The order of traffic signs and frame shapes was randomized to avoid order effects. First, the order of the three traffic scenarios was randomized for each participant (Figure 2, Step 1). Second, within each scenario, the presentation order of the three sign-frame shapes was randomized (Figure 2, Step 2). In contrast, the order of the five warning-intensity levels was consistent. The levels were arranged from left to right in ascending order, from levels 0 to 4. This fixed arrangement was adopted because the participating children were accustomed to left-to-right reading and writing. Therefore, the direction of increasing warning intensity remained consistent throughout the experiment. It helped minimize confusion and errors when the children assigned colors to each warning-intensity level.

To help participants immerse themselves in the context of each sign, before each block, the experiment operator introduced a brief scenario that clarified the viewer’s role. For example, when the task involved “Caution: Pedestrians,” children were invited to imagine they were *drivers* who needed to watch out for people crossing the road; when the task involved “Caution: Motor Vehicles,” they were asked to imagine being *pedestrians* walking near a busy street. This role-switching design aimed to help participants experience the situational meaning of each warning sign from multiple perspectives, thereby supporting a more authentic interpretation of color warning effects. Throughout these transitions, the experiment operator maintained a neutral tone, emphasizing contextual understanding rather than suggesting any specific color choice. Researchers observed and assisted only with technical issues and did not influence participants’ decisions.

*Step 4: semi-structured interviews*: Following the main task, each child participated in a brief semi-structured interview designed to explore the reasoning behind their color and shape choices. The interview aimed to uncover how children perceive the warning function of traffic sign colors, whether shape affects color perception, and which visual features draw their initial attention.

The interviewer used a standard set of questions followed by open-ended conversation to facilitate natural responses while maintaining consistency across participants. The standard questions are listed below:

Q1 Do you recognize these traffic signs? Can you tell me what they mean?

Q2 Why do you think some colors, such as red or yellow, appear to have stronger warning effects?

Q3 When the border shape changes (e.g., triangle, circle, square), do you think it influences your color choice?

Q4 When you first see a traffic sign, what do you notice first: the color, the shape, or the content?

During the subsequent open-ended conversation, children were encouraged to explain their choices in their own words and provide concrete examples where possible. All interviews were audio-recorded and transcribed verbatim for qualitative content analysis in later stages.

*Step 5: task completion and debriefing*: At the end of the experiment, the full panoramic street scene was displayed to participants, showing all of their completed designs. The guide sprite congratulated participants, and small gifts were provided as a token of appreciation for participation.

### 2.4 Data analysis

For the quantitative data analysis, warning intensity was coded on a five-level scale from 0 to 4, corresponding to No warning, Weak warning, Moderate warning, Strong warning, and Very strong warning, as shown in Figure 2. Each color selected by the children was represented in the CIELAB/CIELCH color space by lightness (L*), chroma (C*), and hue angle (*hab*).

In the current within-subject experiment, the same children completed all shape and scenario conditions, the observations were treated as repeated measurements. Since the experimental data comprised both discrete categorical variables (standard color options) and continuous color-space parameters (the possibility for children to freely select colors), the overall distribution did not conform to the classical normality assumption. In particular, the continuous measures conditioned on categorical factors often exhibited multimodal or skewed distributions.

Therefore, for L* and C*, differences among the three shape conditions or the three scenario conditions were examined separately at each warning level using Friedman tests (Friedman, 1937; Hollander et al., 2013). When an omnibus Friedman test indicated a statistically significant difference (*p* ≤ 0.05), paired Wilcoxon signed-rank tests (Hollander et al., 2013) were subsequently conducted to identify the condition pairs responsible for the overall effect.

Furthermore, because *hab* is an angular variable for which 0° and 360° denote the same direction, each hue angle was transformed into a two-dimensional unit vector, (cos *hab*, sin *hab*). Differences among the three within-subject conditions shown in Figure 1 were then evaluated using repeated-measures circular multivariate analysis of variance (MANOVA) (Landler et al., 2022, 2026). In addition, hue observations with *C* *≤ 1 were treated as effectively achromatic and excluded because hue is undefined or numerically unstable near the achromatic axis. When an omnibus circular MANOVA indicated a statistically significant difference (*p* ≤ 0.05), paired two-condition circular MANOVA tests were subsequently conducted to identify the condition pairs responsible for the overall effect.

Moreover, for subsequent distributional visualization, Gaussian kernel density estimation with bandwidth determined according to Scott’s rule (Wêglarczyk, 2018) was used for the linear L* and C* components. While, the distribution of *hab* was visualized using circular kernel density estimation with a von Mises kernel and a circular-data-specific bandwidth selector (Taylor, 2008). The mean hue angle, $\bar{h}$*ab*, was calculated using the circular mean as follows:


$${\bar{h}}_{a\,b}=\mathrm{atan2}\,\left(\frac{1}{n}\,\overset{n}{\underset{i=1}{\sum }}\text{sin}\,h_{a\,b,i},\frac{1}{n}\,\overset{n}{\underset{i=1}{\sum }}\text{cos}\,h_{a\,b,i}\right),$$

where *hab,i* denotes the hue angle of the *i*th observation in radians.

On the other hand, sex was treated as a between-subject factor because boys and girls constituted two mutually exclusive and independent groups. For each combination of traffic scenario, sign shape, and warning-intensity level, the L* and C* values selected by boys and girls were compared using Mann-Whitney U tests (MacFarland and Yates, 2016).

Each hue angle was transformed into a two-dimensional unit vector (cos *hab*, sin *hab*), and sex differences in hue were evaluated using an two-group circular multivariate analysis of variance (MANOVA), with sex specified as the between-subject grouping variable (Landler et al., 2022, 2026).

Overall, the statistical analyses for quantitative data were mainly performed using Python 3.9 with the SciPy, pandas, and NumPy packages (Virtanen et al., 2020; McKinney, 2010; Van Der Walt et al., 2011), while data visualizations were created using Matplotlib (Hunter, 2007).

For the qualitative data analysis, the qualitative data were collected mainly through the semi-structured interviews with participants. The recorded sessions were transcribed and subsequently coded. Based on the coded data, we performed a thematic analysis (Clarke and Braun, 2017) to identify, summarize, and categorize the underlying reasons behind children’s color choices. The coding and thematic analysis were conducted by two additional researchers who were not involved in the qualitative data collection. Each researcher conducted the coding process independently. The analysis involved familiarization with the data, generation of initial codes. Then, they compared the current coding results, discussed any discrepancies and developed a consolidated codebook. Based on this codebook, they independently recoded the interview transcripts, after which inter-coder reliability was assessed using Cohen’s kappa coefficient (Cheung and Tai, 2023). Furthermore, each researcher independently grouped the codes into themes and defined the themes. They then compared and consolidated their findings to produce the final results, thereby minimizing individual researcher bias and enhancing the reliability of the analysis.

## 3 Results

### 3.1 Participant characteristics

Overall, 30 Grade 5 students were recruited from the same local primary school, including 15 boys (age 11, *n* = 12; age 12, *n* = 3) and 15 girls (age 11, *n* = 13; age 12, *n* = 2). All participants had received the mandatory traffic safety education provided by the school.

### 3.2 Results of quantitative analysis

Figure 3 presents the overall color distribution, aggregating children’s selections across all nine frame-icon combinations and their associated warning levels. The selected colors were further analyzed within the CIELCH color space, with lightness (L*), chroma (C*), and hue (*hab*) examined separately to provide a detailed characterization and visualization of the relationship between color and warning level. We then conducted additional statistical analyses.

**FIGURE 3:**
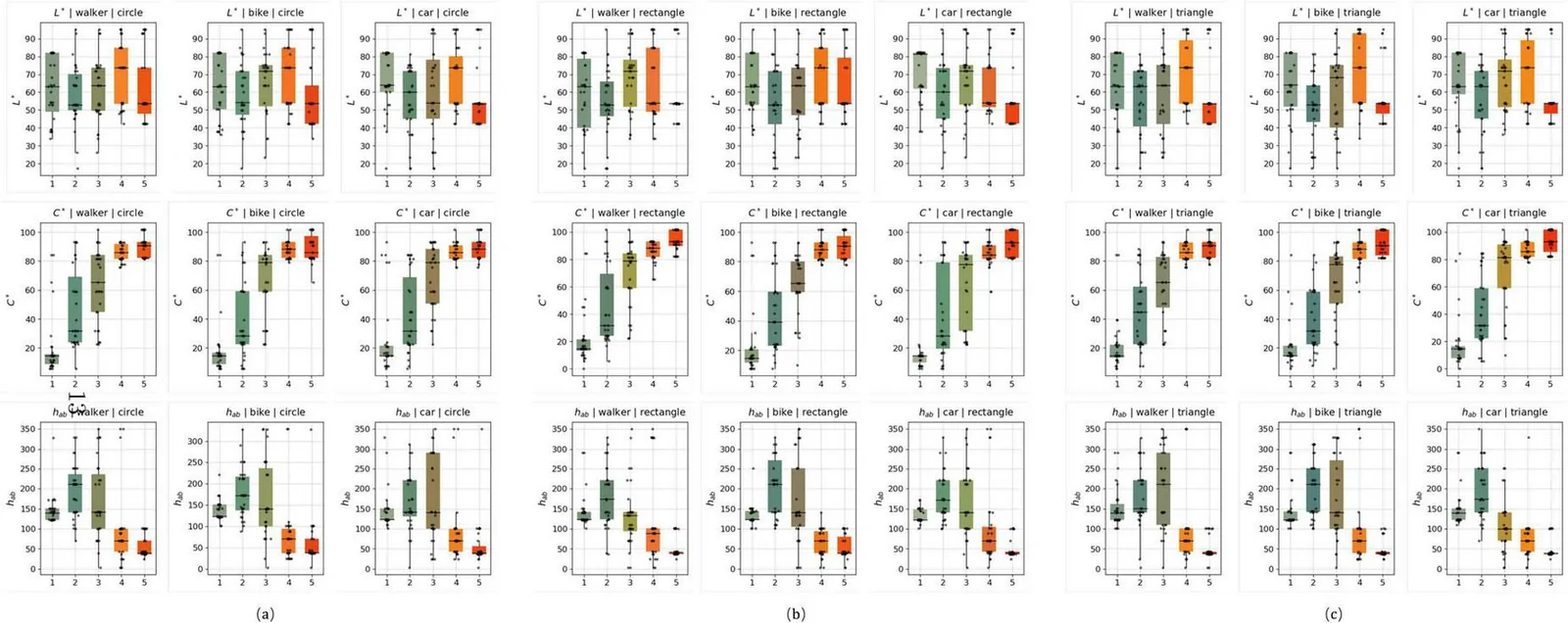
Overall color distributions. **(a–c)** The overall color distributions grouped by shape, including triangle, circle, and rectangle, respectively. Within each panel, the three columns from left to right correspond to the “Caution: Pedestrians,” “Caution: Non-Motor Vehicles,” and “Caution: Motor Vehicles” scenarios, respectively. For each boxplot, the background color represents the mean color of the corresponding group. Each subplot title indicates the associated CIELCH color parameter (L*, C*, or *h*_*ab*_), scenario, and warning-sign shape. The scenario labels walker, bike, and car represent “Caution: Pedestrians,” “Caution: Non-Motor Vehicles,” and “Caution: Motor Vehicles,” respectively.

#### 3.2.1 Sex differences

As discussed in section 3.1, the participant sample was concentrated within a narrow range for most sociodemographic characteristics, except for sex. Therefore, the analysis focused on potential sex differences in children’s warning-color selections across all traffic-safety scenarios and sign-shape conditions. The comparisons were conducted separately for the three CIELCH color components, including L*, C*, and *hab*, corresponding to previous section 2.4. We investigated the mean values and statistical differences as shown in Figure 4. Following the procedures described in section 2.4, Mann-Whitney U tests were used for L* and C*, whereas independent-samples circular MANOVA was used for *hab*. Arithmetic means were calculated for L* and C*, whereas the circular mean was used for *hab*. Even though the mean value variations between male and female factors could be noticed in Figure 4, the results indicated no statistically significant sex differences in color selections for most combinations of traffic scenario and sign shape across the five warning-intensity levels.

**FIGURE 4:**
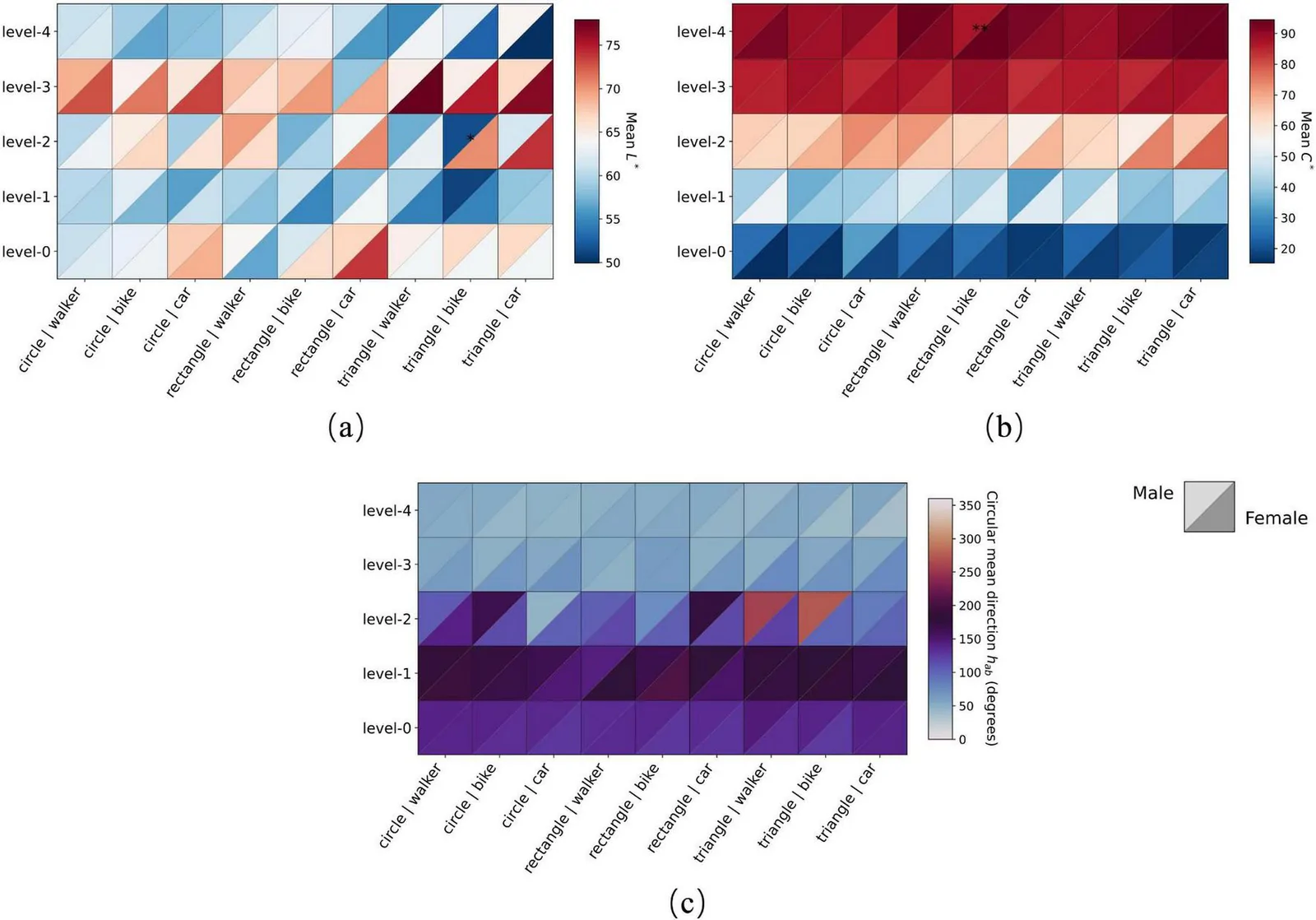
Sex differences in warning-color perception across traffic-safety scenarios and sign shapes. **(a–c)** Present L*, C*, and *h*_*ab*_, respectively. Within each rectangular box, the upper and lower triangles represent boys and girls, respectively. Arithmetic means are shown for L* and C*, whereas circular means are shown for *h*_*ab*_. Sex differences were evaluated using Mann-Whitney U tests for L* and C* and independent-samples circular MANOVA for *h*_*ab*_. Statistical significance is indicated by asterisks: *p ≤ 0.05 and **p ≤ 0.01.

#### 3.2.2 Comparison of shape effects within the same scenario

In this section, we examined whether shape influenced children’s selection of warning colors within the same scenario. Specifically, we initially focused on the “Caution: Motor Vehicles” condition, which represents a typical traffic warning sign that children frequently encounter on their way home from school. In this scenario, the warning indicates that the observer (pedestrian) may be at risk of injury from approaching motor vehicles. Within this controlled scenario, children were asked to assign colors to fill in the choosen border shape of the warning sign (circle, triangle, or square) corresponding to warning levels (0–4).

To evaluate potential differences among the three shape conditions, comparisons were conducted separately for each warning-intensity level and CIELCH component. Friedman tests were used for L* and C*. For *hab*, the hue angles were transformed into two-dimensional unit vectors (cos *hab*, sin *hab*), and analyzed using repeated-measures circular MANOVA. A summary of the results is presented in Table 1.

**TABLE 1:** Comparison of shape effects under the “Caution: Motor Vehicles” scenario using Friedman tests for L* and C* and repeated-measures circular MANOVA for *h*_*ab*_.

Level	L*			C*			*hab*	
	*Q-stat*	*p*-value	Kendall’s *W*	*Q-stat*	*p*-value	Kendall’s *W*	Pillai-trace	*p*-value
Level-0	1.705	0.426	0.027	4.345	0.114	0.070	0.094	0.224
Level-1	5.212	0.074	0.084	0.835	0.659	0.013	0.048	0.572
Level-2	4.384	0.112	0.071	2.240	0.326	0.036	0.181	0.020*
Level-3	3.020	0.221	0.049	4.489	0.106	0.072	0.073	0.353
Level-4	0.333	0.846	0.005	13.872	0.001***	0.224	0.062	0.451

For L* and C*, *Q*-stat denotes the Friedman test statistic and Kendall’s W the corresponding effect size. For *h*_*ab*,_ Pillai-trace denotes the repeated-measures circular MANOVA statistic. Statistical significance is indicated by asterisks: *p ≤ 0.05 and **p ≤ 0.01.

As shown in the results presented in Table 1, we conducted a total of 15 comparisons across three color dimensions (L*, C*, and *hab*) and five warning levels (0–4). A significance threshold of *p* ≤ 0.05 was adopted, with significant results denoted by an asterisk (*). Overall, the analyses revealed no substantial evidence that shape influences children’s perception of warning colors, as 13 out of the 15 tests yielded *p*-values greater than 0.05. Only two significant differences were observed: one in C* at warning level 4 (*p* = 0.001) and another in *hab* at warning level 2 (*p* = 0.020). To identify the condition pairs responsible for these effects, post hoc comparisons were conducted using paired Wilcoxon signed-rank tests for C* and paired two-condition circular MANOVA tests for *hab*. The results are reported in Tables 2, 3, respectively.

**TABLE 2:** Pairwise Wilcoxon signed-rank tests of C * distributions across shapes at warning level 4 in the “Caution: Motor Vehicles” scenario.

Level	Shape 1	Shape 2	*W*-stat	*p*-value
Level-4	Circle	Rectangle	70.5	0.116
Level-4	Circle	Triangle	38.0	0.004**
Level-4	Rectangle	Triangle	14.5	0.099

*W*-stat denotes the Wilcoxon signed-rank test statistic. Statistical significance is indicated by asterisks: **p ≤ 0.01.

**TABLE 3:** Pairwise two-condition circular MANOVA tests of *h*_*ab*_ distributions across shapes at warning level 2 in the “Caution: Motor Vehicles” scenario.

Level	Shape 1	Shape 2	Pillai-trace	*p*-value
Level-2	Circle	Rectangle	0.115	0.169
Level-2	Circle	Triangle	0.184	0.052
Level-2	Rectangle	Triangle	0.206	0.035*

Statistical significance is indicated by asterisks. *p ≤ 0.05.

Furthermore, because risk perception could differ depending on whether the potential victim is oneself or others (Globig et al., 2022), we further examined two additional traffic warning scenarios. The first was the “Caution: Pedestrians” condition, in which the hazard is directed at pedestrians and thus primarily threatens others instead of oneself (self as the vehicle driver). The second was the “Caution: Non-Motor Vehicles” condition, in which the perceived threat is more nuanced: the potential victim could be oneself (as a pedestrian exposed to cyclists and other non-motor vehicles) or others (when imagining oneself as a motor vehicle driver posing risk to non-motor vehicle users).

For these two scenarios, we applied the same analytical procedure as in the ‘Caution: Motor Vehicles’ condition. Friedman tests were used to examine overall differences in L* and C* across the three shape conditions, followed, when statistically significant, by pairwise Wilcoxon signed-rank tests. Differences in *hab* were examined using repeated-measures circular MANOVA, followed, when statistically significant, by paired two-condition circular MANOVA tests. The results for the “Caution: Pedestrians” and “Caution: Non-Motor Vehicles” scenarios are summarized in Tables 4, 5, respectively.

**TABLE 4:** Comparison of shape effects under the “Caution: Pedestrians” scenario using Friedman tests for L* and C* and repeated-measures circular MANOVA for *h*_*ab*_.

Level	L*			C*			*hab*	
	*Q-*stat	*p*-value	Kendall’s *W*	*Q-*stat	*p*-value	Kendall’s *W*	Pillai-trace	*p*-value
Level-0	0.306	0.858	0.005	2.282	0.319	0.037	0.056	0.510
Level-1	0.780	0.677	0.013	1.061	0.588	0.017	0.032	0.747
Level-2	1.021	0.600	0.016	1.213	0.545	0.020	0.123	0.103
Level-3	0.532	0.766	0.009	1.909	0.385	0.031	0.095	0.207
Level-4	1.101	0.577	0.018	3.341	0.188	0.054	0.039	0.670

The statistical tests, reported statistics, and significance notation are the same as those used in Table 1.

**TABLE 5:** Comparison of shape effects under the “Caution: Non-Motor Vehicles” scenario using Friedman tests for L* and C* and repeated-measures circular MANOVA for *h*_*ab*_.

Level	L*			C*			*hab*	
	*Q*-stat	*p*-value	Kendall’s *W*	*Q*-stat	*p*-value	Kendall’s *W*	Pillai-trace	*p*-value
Level-0	0.791	0.673	0.013	2.891	0.236	0.047	0.076	0.319
Level-1	3.881	0.144	0.063	0.540	0.763	0.009	0.082	0.282
Level-2	3.840	0.147	0.062	0.772	0.680	0.012	0.019	0.888
Level-3	2.412	0.299	0.039	1.806	0.405	0.029	0.055	0.494
Level-4	4.081	0.130	0.066	2.675	0.262	0.043	0.053	0.514

The statistical tests, reported statistics, and significance notation are the same as those used in Table 1.

As shown in Tables 4, 5, changes in the scenario and the perceived target of danger (i.e., whether the risk was directed toward oneself or others) had little influence on the relationship between sign shape and color choice. Across both scenarios, a total of 3 × 5 × 2 group comparisons were conducted, and all yielded *p*-values greater than 0.05.

#### 3.2.3 Comparison of scenario effects within the same shape

In this section, we examined whether different scenarios influenced children’s selection of warning colors when the shape was held constant. Following international standards (the Vienna Convention on Road Signs and Signals and ISO 7010) (International Organization for Standardization, 2019) and Chinese standards (GB 5768.2-2022 and GB 2894-2025) (Standardization Administration of China, 2022, 2025), the triangle was taken as the baseline shape for warning-sign design. The analytical procedure used here was consistent with that described in Section 3.2.2.

A summary of the scenario-effect analyses under the triangle condition is presented in Table 6. Twelve of the 15 omnibus comparisons yielded no statistically significant differences. Significant scenario effects were observed only for C* at warning level 0 (*p* = 0.024), L* at warning level 2 (*p* = 0.016), and *hab* at warning level 2 (*p* = 0.029).

**TABLE 6:** Comparison of scenario effects under the triangle shape using Friedman tests for L* and C* and repeated-measures circular MANOVA for *h*_*ab*_.

Level	L*			C*			*hab*	
	*Q*-stat	*p*-value	Kendall’s *W*	*Q*-stat	*p*-value	Kendall’s *W*	Pillai-trace	*p*-value
Level-0	1.918	0.383	0.031	7.485	0.024*	0.121	0.079	0.317
Level-1	1.788	0.409	0.029	0.533	0.766	0.009	0.007	0.982
Level-2	8.234	0.016*	0.133	3.945	0.139	0.064	0.171	0.029*
Level-3	0.141	0.932	0.002	2.156	0.340	0.035	0.027	0.805
Level-4	0.372	0.830	0.006	6.094	0.051	0.092	0.033	0.735

The reported statistics and significance notation are the same as those used in Table 1.

Follow-up pairwise analyses were conducted to identify the scenario pairs associated with these overall effects. For C* at warning level 0, none of the pairwise Wilcoxon signed-rank tests reached statistical significance despite the significant omnibus result (Table 7). Therefore, this overall effect could not be attributed to a specific pair of scenarios. At warning level 2, L* differed between the “Caution: Motor Vehicles” scenario and both the “Caution: Pedestrians” and “Caution: Non-Motor Vehicles” scenarios (*p* = 0.043 and *p* = 0.046, respectively; Table 8). Similarly, the pairwise circular MANOVA for *hab* at warning level 2 identified a significant difference only between the “Caution: Pedestrians” and “Caution: Motor Vehicles” scenarios (*p* = 0.004; Table 9). Overall, scenario effects were limited and occurred primarily in comparisons involving the “Caution: Motor Vehicles” scenario, indicating that children’s warning-color selections were generally stable across the three traffic scenarios under the current triangle sign shape context.

**TABLE 7:** Pairwise Wilcoxon signed-rank tests of C* distributions across scenarios at warning level 0 under the triangle shape.

Level	Scenario 1	Scenario 2	*W*-stat	*p*-value
Level-0	Walker	Bike	162.0	0.989
Level-0	Walker	Car	87.5	0.074
Level-0	Bike	Car	76.0	0.101

**TABLE 8:** Pairwise Wilcoxon signed-rank tests of L* distributions across scenarios at warning level 2 under the triangle shape.

Level	Scenario 1	Scenario 2	*W*-stat	*p*-value
Level-2	Walker	Bike	167.5	0.605
Level-2	Walker	Car	114.0	0.043*
Level-2	Bike	Car	115.5	0.046*

Statistical significance is indicated by asterisks: *p ≤ 0.05.

**TABLE 9:** Pairwise two-condition circular MANOVA tests of *h*_*ab*_ distributions across scenarios at warning level 2 under the triangle shape.

Level	Scenario 1	Scenario 2	Pillai-trace	*p*-value
Level-2	Walker	Bike	0.042	0.539
Level-2	Walker	Car	0.322	0.004**
Level-2	Bike	Car	0.150	0.095

Statistical significance is indicated by asterisks: **p ≤ 0.01.

In addition, we performed the same set of analyses for the other two common warning sign shapes, the circle and the square. The results are summarized in Tables 10, 11. Across all warning levels, and for each of the three color-space dimensions (L*, C*, and *hab*), children’s color selections showed no significant differences among the three scenarios, as all *p*-values were greater than or substantially above the 0.05 threshold. Thus, when the warning sign shape was fixed as a circle, the selection of warning colors remained consistent across scenarios, and the same conclusion held when the shape was fixed as a rectangle.

**TABLE 10:** Comparison of scenario effects under the circle shape using Friedman tests for L* and C* and repeated-measures circular MANOVA for *h*_*ab*_.

Level	L*			C*			*hab*	
	*Q*-stat	*p*-value	Kendall’s *W*	*Q*-stat	*p*-value	Kendall’s *W*	Pillai-trace	*p*-value
Level-0	1.402	0.496	0.023	2.353	0.308	0.038	0.086	0.255
Level-1	2.509	0.285	0.040	3.189	0.203	0.051	0.074	0.336
Level-2	2.752	0.253	0.044	0.860	0.651	0.014	0.049	0.562
Level-3	0.258	0.879	0.004	4.822	0.090	0.078	0.014	0.929
Level-4	0.217	0.897	0.003	3.610	0.164	0.058	0.041	0.648

The reported statistics and significance notation are the same as those used in Table 1.

**TABLE 11:** Comparison of scenario effects under the rectangle shape using Friedman tests for L* and C* and repeated-measures circular MANOVA for *h*_*ab*_.

Level	L*			C*			*hab*	
	*Q*-stat	*p-*value	Kendall’s *W*	Q-stat	*p*-value	Kendall’s *W*	Pillai-trace	*p-*value
Level-0	4.356	0.113	0.070	2.854	0.240	0.046	0.049	0.569
Level-1	1.750	0.417	0.028	1.212	0.546	0.020	0.069	0.375
Level-2	4.297	0.117	0.069	1.940	0.379	0.031	0.097	0.200
Level-3	0.614	0.736	0.010	3.361	0.186	0.054	0.087	0.250
Level-4	0.966	0.617	0.016	1.101	0.577	0.018	0.023	0.841

The reported statistics and significance notation are the same as those used in Table 1.

#### 3.2.4 Warning color selection for children

This study selected four representative traffic-sign colors as benchmark colors for the following discussion. These four colors were defined with reference to the Japanese Industrial Standards (JIS) for safety colors. The corresponding sRGB and CIELCH coordinates are summarized below. We treated these data as representative adult-oriented reference values for traffic-sign-related warning colors.

- Red: sRGB = (251, 28, 42); CIELCh: L* = 53.51, C* = 93.07, *hab* = 34.15°.
- Yellow: sRGB = (255, 217, 0); CIELCh: L* = 87.43, C* = 87.53, *hab* = 91.92°.
- Blue: sRGB = (11, 73, 157); CIELCh: L* = 32.41, C* = 53.07, *hab* = 287.35°.
- Green: sRGB = (1, 115, 86); CIELCh: L* = 42.67, C* = 36.37, *hab* = 166.77°.

Furthermore, building upon the results in sections 3.2.2 and 3.2.3, we confirmed that children’s choices of warning colors in traffic signs are largely unaffected by the scenario or the shape of the sign. Therefore, we integrated all samples across scenarios and shapes to examine children’s selections of warning colors for traffic signs across different warning levels (from levels 0 to 4).

First, although children were instructed to select colors corresponding to different warning levels, their responses revealed consistent trends at the upper end of the scale. For the highest warning level (very strong warning or prohibition), children overwhelmingly chose red. This is consistent with the interpretation that prohibition represents the most severe warning, even though in national standards red is generally defined as prohibitory rather than cautionary. In contrast, yellow is typically used in traffic safety to convey warnings, and in our data, children tended to associate yellow with level 3 (strong warning), as observed from Figure 3.

Despite this general alignment, the CIELCH analysis indicated noticeable differences between children’s color selections and the standard reference values, as shown in Figure 3. To further examine these distributional patterns, kernel density estimation (KDE) was applied separately according to the characteristics of each color component. For the linear L* and C* components, Gaussian KDE was used, with the bandwidth determined according to Scott’s rule (Wêglarczyk, 2018). Because *hab* is an angular variable, its distribution was estimated using circular KDE with a von Mises kernel and a circular-data-specific bandwidth selector (Taylor, 2008). Circular means, rather than arithmetic means, were used to summarize the central tendency of *hab*. These methods accommodate the irregular and potentially multimodal distributions arising from the combination of predefined and freely selected colors while preserving the circular structure of hue.

As shown in Figure 5, the KDE-based density curves were complemented by the calculation of mean L*, C*, and *hab* values of children’s selections, with the corresponding standard-defined values indicated for comparison. The results revealed that children’s hue selections (*hab*) at the highest warning level (very strong warning or prohibition) were broadly consistent with the standard reference for red, exhibiting a unimodal distribution concentrated near the reference value. At warning level 3, corresponding to yellow, the circular mean of *hab* deviated from the reference value, while the peak of the skewed distribution still remains close to it.

**FIGURE 5:**
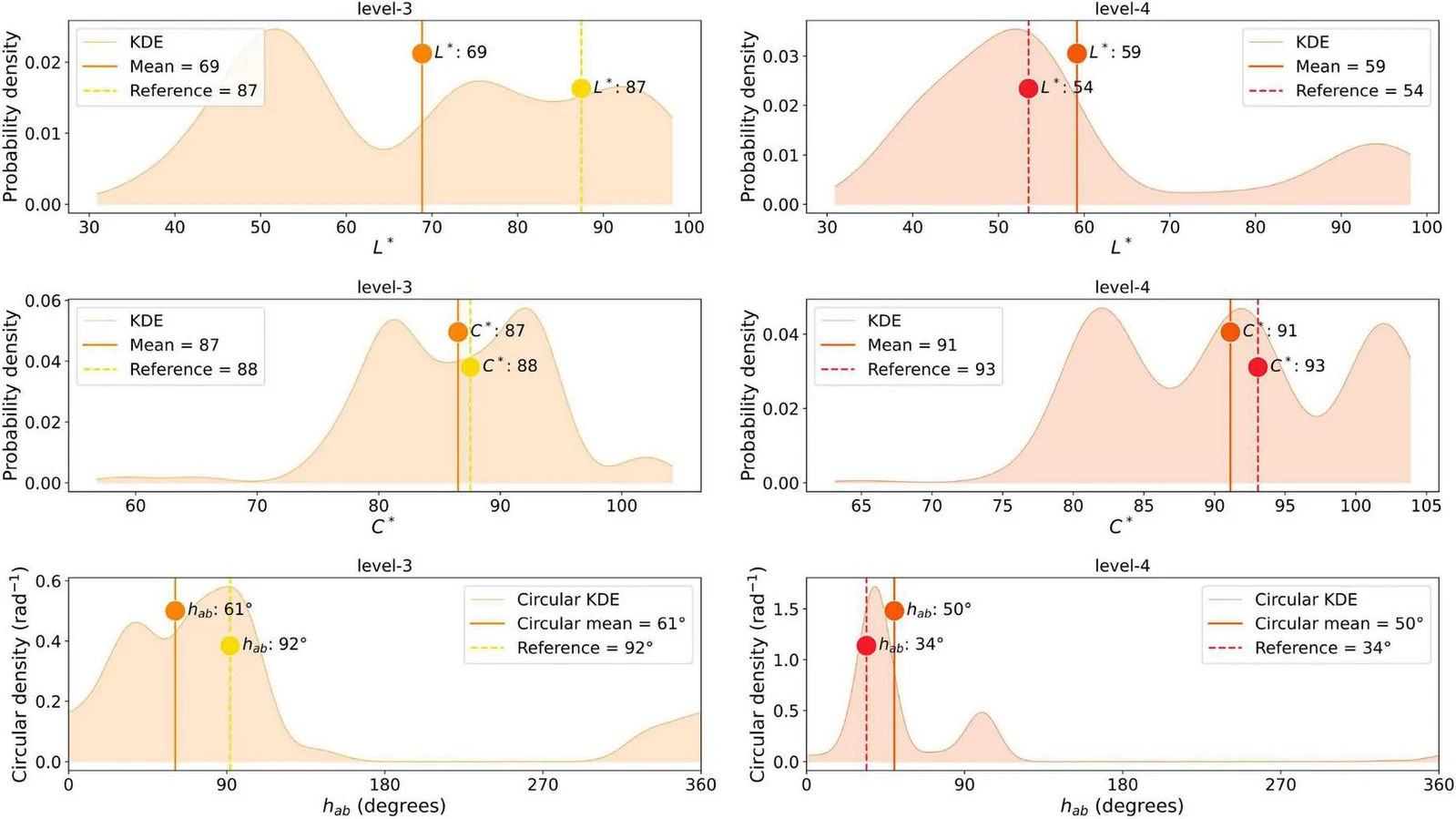
CIELCH color-space distributions of children’s color selections at warning levels 3 and 4. Gaussian KDE was used for L* and C*, whereas circular KDE with a von Mises kernel was used for *h*_*ab*_. The filled density curves show the estimated distributions; the solid lines and circular markers indicate the arithmetic means for L* and C* and the circular mean for *h_*ab*_*. The dashed lines indicate the standard reference values for yellow and red.

Furthermore, children’s chroma (C*) responses exhibited clear multimodality, although the mean values remained close to the standardized reference values. As shown in Figure 5, a substantial proportion of the distributions was concentrated in regions with higher chroma values.

More substantial deviations from the standardized reference values were observed for lightness (L*). At both levels 3 and 4, the mean L* values differed noticeably from their corresponding reference values. At level 3, which corresponded to yellow, the distribution was multimodal: although its mean was lower than the reference value, the higher-L* peak was located near that value. At level 4, the mean L* was considerably higher than the standardized red reference value, and a secondary peak occurred in an even higher-lightness region.

##### 3.2.4.1 Summary of hypothesis testing

Taken together, H1 was not supported overall. Sign shape produced only limited and isolated differences in children’s warning-color selections at specific warning levels and color dimensions, rather than a consistent effect across conditions. H2 was likewise not supported overall, because children’s color selections remained largely stable across the three pictorial scenarios, with only two isolated difference observed for hue at warning level 2 under the triangle condition. H3 received partial support. Children’s hue selections at warning level 4 broadly corresponded with the adult-oriented standardized reference value for red. At warning level 3, the circular mean hue deviated from the reference value for yellow, although the peak of the skewed distribution remained close to it. Children’s chroma distributions at both warning levels were heterogeneous and multimodal, while their lightness values showed clearer deviations from the corresponding standardized reference values.

### 3.3 Results of qualitative analysis

As described in section 2.4, Cohen’s kappa coefficient was 0.82, indicating a high level of inter-coder agreement. In summary, the resulting qualitative analysis revealed a consistent pattern: color functioned as the primary perceptual cue in children’s assignment of warning intensity, and their color choices remained largely stable across sign shapes and traffic scenarios. The percentages reported below were calculated using the number of children who provided relevant responses to each question or coding category as the denominator.

When explicitly asked which element they noticed first (Q4), 11 of the 30 children identified color, 6 identified shape, and 13 prioritized sign content. These findings indicate that children attended to multiple visual channels, including color, shape, and pictorial meaning. However, the element noticed first did not necessarily determine the color selected for a given warning-intensity level.

Across the dataset, in the conversations related to Q2, red was the most frequently named color (19 of the 30 children), and *yellow* was also commonly named (9 children). More broadly, 22 children used descriptors associated with perceptual salience, such as *bright, vivid*, and *eye-catching.* These expressions suggest that perceived visibility played an important role in children’s color choices, although the possible influence of their knowledge of traffic conventions cannot be excluded. For example, one representative response was, “*I chose red and yellow because they are brighter, and people notice them more easily.*” Another was, “*It is brighter, so it is easier to see.*” These qualitative responses further support the quantitative finding that sign shape and scenario generally had limited effects on children’s warning-color selections. Overall, children appeared to associate effective warning colors with high visual salience and ease of detection.

Furthermore, during the conversations corresponding to Q3, many children recognized the conventional meanings associated with sign shapes. In particular, the triangle was described as *more warning-like* by 10 of the 30 children. However, this recognition did not systematically change the colors they assigned to signs with high warning intensities. Eight children explicitly reported using the same color across different frame shapes. For instance, one child explained that they had chosen red for both the circular and triangular frames: “*This circle is red, and I also chose red for that triangle.*” In other words, shape may modulate perceived urgency, but it does not reliably alter a child’s color choice at a given warning-intensity level.

Moreover, across the responses to Q2, Q3, and Q4, 18 of the 30 children directly mentioned scenario-related or environmental factors. However, these factors did not appear to be dominant determinants of color choice. Among these 18 children, only 2 specifically stated that background lighting or surrounding vegetation would lead them to select a different color. More commonly, 13 of the 18 children used contextual reasoning to explain why a bright color would be effective in particular settings. In short, contextual factors were discussed primarily as modifiers of visibility rather than as the principal basis for color selection.

However, several exceptions to the general pattern emerged during the overall qualitative analysis. Half of the sample (15 of the 30 children) explicitly stated that shape could influence their color choices in some cases. For example, some suggested that a triangular frame *should be red*, whereas a square might appear less salient. A few participants also made individual color selections that differed from the general pattern, such as darker red, pinkish variants, or orange for particular frames. These exceptions suggest that shape and scenario can act as secondary factors that modify hue, chroma, or lightness choices for some children. Nevertheless, these responses do not overturn the general tendency for children to choose visually salient colors. They may also help explain the isolated effects observed in the preceding analyses of shape and scenario influences on warning-color selection.

Taken together, the qualitative evidence supports the conclusion that children’s choices of warning colors are governed principally by perceptual salience (brightness, vividness, and contrast) and are therefore largely independent of sign shape and immediate scene context. Shape and context instead operate primarily as reinforcing or modulating cues rather than as primary determinants. This interpretation reconciles two observable facts in the dataset: (a) children readily recognized and named shape conventions, especially the triangle as a warning sign; and (b) the color assignments they produced for high warning levels remained overwhelmingly oriented toward high-salience hues, especially red, irrespective of shape or scene.

## 4 Discussion

This study provides evidence of how primary-school-aged children associate different colors with varying levels of warning intensity. These findings could inform the initial design and evaluation of child-oriented traffic warning signs and provide a basis for further studies in comparable cultural and educational contexts.

First, the quantitative results indicate that children’s warning-color selections were largely robust to variations in sign shape (triangle, circle, rectangle) and scenario (“Caution: Pedestrians,” “Caution: Non-Motor Vehicles,” and “Caution: Motor Vehicles”), with only a few isolated effects at specific warning levels and color parameters. Across conditions, red was consistently chosen for the highest warning level and yellow for strong warnings, broadly aligning with conventional safety-color semantics. These findings were generally consistent with adult-oriented color standards (Gopang et al., 2024). However, compared with those primarily adult-oriented standards, our study revealed deviations in lightness and chroma. At the highest warning-intensity level (level 4), children selected colors with higher L* values, indicating greater lightness, than the corresponding reference value. Moreover, the distributions of L* at level 3 and C* at levels 3 and 4 were multimodal, indicating heterogeneous color selections among the children. Second, the qualitative findings indicate that color functions as the primary cue for conveying warning intensity, whereas shape and scenario serve mainly as secondary, modulating factors. Children typically justified their choices in terms of visibility and salience rather than formal traffic rules.

While adults typically interpret traffic signs as an integrated whole by combining shape, icon information, and color to understand both meaning and warning intensity (Prada et al., 2016; Ng and Chan, 2008), the findings of this study suggest that children use a different perceptual strategy in the context of traffic-safety sign design. For children, the meaning communicated by the icon (for example, a car indicating a possible hazard) and the degree of warning intensity appear to be treated as two separate aspects. Through the shape and content of a sign, children can recognize that a dangerous situation may occur and that they need to pay attention. This interpretation aligns with previous studies (Waterson and Monk, 2014; Siu et al., 2015). However, the perceived level of danger, reflected in warning intensity, does not appear to depend strongly on icon content or frame shape. Instead, it relies more heavily on color itself. In other words, other visual design elements, including frame shape and pictorial content, do not substantially increase the warning intensity perceived by children. More importantly, according to the mandatory educational requirements and as confirmed by teachers at the primary school from which the participants were recruited, the Grade 5 children had already received basic education on traffic signs, including their key visual elements, such as color, shape, and pictorial content (State Council of China, 2007). Given that sensitivity to color emerges early in infancy (Skelton et al., 2022), the present findings suggest that, in the context of judging traffic sign warning intensity, color may still function as a more immediate and perceptually salient cue for children than sign shape or pictorial content, even considering the educational background.

### 4.1 Limitations and future work

Several limitations should be considered when interpreting the findings of this study. First, the present study cannot directly distinguish the influence of intrinsic perceptual salience from that of learned educational associations. All participants had received basic public safety education. Second, although all child participants were recruited from local families within the primary school’s catchment area and shared relatively similar cultural backgrounds, differences in their families’ educations could not be fully controlled. Third, the sample consisted of Grade 5 students from the primary school in Asia who shared similar educational and cultural backgrounds. Consequently, the findings may not generalize directly to children at different stages of cognitive development, or children living in other cultural and regulatory environments. Fourth, although the total time required to complete all 45 tasks was similar across child participants (approximately 15 min), the time spent on each individual task was not recorded. Consequently, the results cannot reveal whether task completion time influenced participants’ final perceptions of color warning intensity.

Future research should examine the respective contributions of perceptual salience and learned associations through more tightly controlled developmental and cross-cultural studies while also collecting detailed task-level completion-time data. Furthermore, children from different age groups, regions, cultural backgrounds, and family educations could be recruited to determine whether children’s perceptions of color-based warning intensity remain stable across these factors.

## 5 Conclusion

This study investigated how children perceive color-based warning intensity in traffic-safety signs and whether their perceptions are influenced by sign shape and pictorial scenario. A gamified color-selection task and semi-structured interviews were conducted with 30 children. Overall, the findings indicate that children rely primarily on color to judge warning intensity, whereas sign shape and pictorial scenario function mainly as secondary contextual cues. The study therefore provides child-centered empirical evidence for the design and evaluation of traffic-safety signs. The main contributions of this study are summarized as follows:

(1) The sign shape has limited and mostly isolated effects on children’s warning-color selections. Shape therefore appears to function as a secondary or reinforcing cue rather than as a primary determinant of perceived warning intensity.

(2) Children’s warning-color selections remained largely consistent across different pictorial traffic scenarios. This finding indicates that scenario context has limited systematic influence on children’s color selections, whereas color plays a more central role in conveying warning intensity.

(3) Children’s associations of red with the highest warning level and yellow with strong warnings broadly corresponded with adult-oriented safety-color definitions. However, their lightness and chroma preferences differed from the standardized values. Thus, within the traffic safety context, although adult-oriented standards provide a useful baseline, it is meaningful to further evaluate and adjust the lightness and chroma of warning colors when designing traffic-safety signs for children.

## Data Availability

The data supporting the findings of this study are available from the corresponding author upon reasonable request.
